# R-Loops in Proliferating Cells but Not in the Brain: Implications for AOA2 and Other Autosomal Recessive Ataxias

**DOI:** 10.1371/journal.pone.0090219

**Published:** 2014-03-17

**Authors:** Abrey J. Yeo, Olivier J. Becherel, John E. Luff, Jason K. Cullen, Thidathip Wongsurawat, Piroon Jenjaroenpoon, Vladimir A. Kuznetsov, Peter J. McKinnon, Martin F. Lavin

**Affiliations:** 1 QIMR Berghofer Medical Research Institute, Radiation Biology and Oncology Laboratory, Brisbane, Queensland, Australia; 2 School of Medicine, University of Queensland, Herston, Queensland, Australia; 3 School of Chemistry and Molecular Biology, University of Queensland, St. Lucia, Queensland, Australia; 4 Department of Genome and Gene Expression Data Analysis, Bioinformatics Institute, Singapore, Singapore; 5 School of Computer Engineering, Nanyang Technological University, Singapore, Singapore; 6 Department of Genetics and Tumour Cell Biology, St. Jude Children's Research Hospital, Memphis, Tennessee, United States of America; University of Tasmania, Australia

## Abstract

Disruption of the *Setx* gene, defective in ataxia oculomotor apraxia type 2 (AOA2) leads to the accumulation of DNA/RNA hybrids (R-loops), failure of meiotic recombination and infertility in mice. We report here the presence of R-loops in the testes from other autosomal recessive ataxia mouse models, which correlate with fertility in these disorders. R-loops were coincident in cells showing high basal levels of DNA double strand breaks and in those cells undergoing apoptosis. Depletion of *Setx* led to high basal levels of R-loops and these were enhanced further by DNA damage both *in vitro* and *in vivo* in tissues with proliferating cells. There was no evidence for accumulation of R-loops in the brains of mice where *Setx*, *Atm*, *Tdp1* or *Aptx* genes were disrupted. These data provide further evidence for genome destabilization as a consequence of disrupted transcription in the presence of DNA double strand breaks arising during DNA replication or recombination. They also suggest that R-loop accumulation does not contribute to the neurodegenerative phenotype in these autosomal recessive ataxias.

## Introduction

The autosomal recessive cerebellar ataxias (ARCAs) are a diverse group of disorders arising from defects in genes involved in the response to DNA damage; mitochondrial function and those controlling different levels of metabolic and other cellular processes [1], [2]. These are a class of progressive neurodegenerative diseases that result from cerebellar atrophy and spinal tract dysfunction [3]. A subgroup of these are characterised by defects in proteins that recognise and/or repair various forms of damage to DNA [4], [5]. The best characterised of these is ataxia-telangiectasia (A-T) which arises due to mutations in the ATM gene [6]. ATM is recruited to DNA double strand breaks (DSB) by the Mre11/Rad50/NBN (MRN) complex where it is activated to phosphorylate a multitude of proteins involved in the response to DNA damage [7]. Disorders arising due to mutations in members of the MRN complex are also characterised by defects in the response to DNA DSB [8]. Hypomorphic mutations in Mre11 give rise to A-T like disorder (ATLD), which overlaps in its clinical phenotype with A-T and also features radiosensitivity and cell cycle defects [9]. Nijmegen breakage syndrome (NBS) is caused by mutations in NBN and is characterised by microcephaly, cell cycle checkpoint defects and ionizing radiation sensitivity [10]. Mutation in the third member of the MRN complex, Rad50, has been reported for a single patient who has an NBS-like disorder as well as a defect in the response to DNA DSB [11], [12]. Failure to resolve DNA single strand breaks (SSB) is also associated with a number of cerebellar atrophies [13] and these include ataxia oculomotor apraxia type 1 (AOA1) and spinocerebellar ataxia with axonal neuropathy (SCAN1). AOA1 is an autosomal recessive cerebellar ataxia syndrome that lacks the extraneurological features of A-T and related disorders [14]. The protein defective in AOA1, aprataxin, resolves abortive DNA ligation intermediates as part of the process of repair of DNA SSB [15], [16]. Mutations in another gene, tyrosyl DNA phosphodiesterase 1 (TDP1) gives rise to SCAN1. TDP1 removes the Topoisomerase (Topo1) complex from DNA terminii primarily at DNA SSB that arise due to collision of the transcription machinery with Topo1 intermediates or due to oxidative stress [17]. Disruption of this gene in mice leads to age-dependent cerebral atrophy and neurons from *Tdp1^−/−^* cells fail to rapidly repair DNA SSB at Topo1 complexes [18]. Another member of this group, ataxia oculomotor apraxia type 2 (AOA2) is also characterised by sensitivity to DNA damaging agents [19], [20]. However, the genomic instability that occurs in AOA2 cells appears to result from the accumulation of DNA/RNA hybrids (R-loops) following collisions between the transcription apparatus and DNA replication forks [21]. In addition, evidence for a role in transcriptional regulation which could also impact on genomic stability has also been reported for senataxin [22]. Recently, we generated the first *Setx* knockout mouse model to investigate the physiological role of senataxin. *Setx^−/−^* mice are defective in spermatogenesis, meiotic recombination and meiotic sex chromosome inactivation [23]. DNA DSBs persist in *Setx^−/−^* spermatocytes as well as R-loops, which appear to collide with Holiday junctions, thus preventing crossing-over. Skourti-Stathaki et al 2011 demonstrated that senataxin resolves R-loops formed at transcriptional pause sites to enable transcription initiation and termination [24]. This is in agreement with previous data providing evidence for transcription readthrough and defects in RNA splicing in senataxin-depleted cells [22]. The yeast ortholog of senataxin, Sen1, has also been shown to resolve R-loops to protect the genome against transcription-associated instability [25]–[28]. R-loops constitute a novel trigger for genomic instability and the accumulation of these structures may represent an underlying and contributing mechanism in autosomal recessive ataxias characterised by defective responses to DNA damage. Accumulating evidences have shown correlations between transcription deregulation, defective RNA processing, genome instability and neurodegeneration [29], [30]. Here, we investigated for the presence of R-loops in both proliferating and non-proliferating tissues in order to address the potential role of R-loops in the neuropathology in autosomal recessive cerebellar ataxias.

## Results

### Accumulation of R-loops in germ cells of ARCA mouse models correlates with infertility

**Figure 1:**
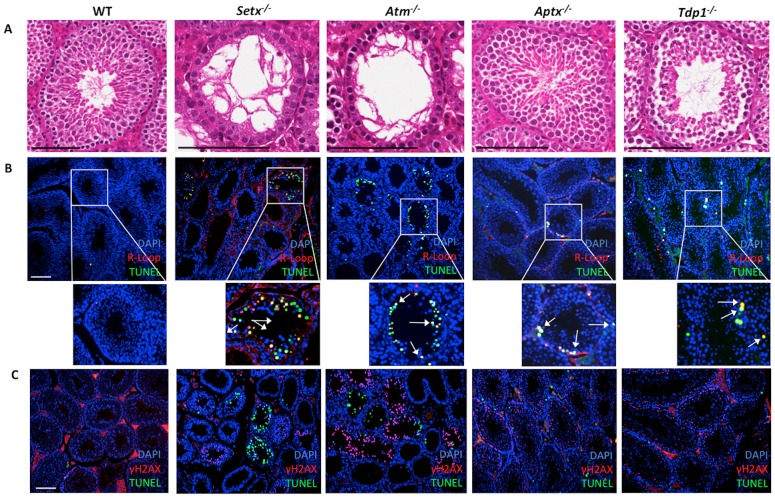
Evidence of DNA/RNA hybrids (R-loops) accumulation in spermatocytes of ARCA mouse model. **A.** Histological cross-sections of seminiferous tubules from ARCA mouse models *Setx^−/−^*, *Atm^−/−^*, *Aptx^−/−^*, *Tdp1^−/−^* and wildtype (WT) littermates. Sections were H & E stained. Scale bar, 100 µm. **B.** Immunostaining of testes sections with R-loop (S9.6) antibody (red) and TUNEL (green). Nuclei were stained using Hoechst 33342 (blue). Scale bar, 100 µm. Magnified views of the tubules are also shown. Representative spermatocytes staining positive for both R-loop and TUNEL are indicated by white arrows. **C.** Immunostaining of testes sections with γH2AX (red), a marker of DNA DSBs, and TUNEL (green). Scale bar, 100 µm.

**Figure 2:**
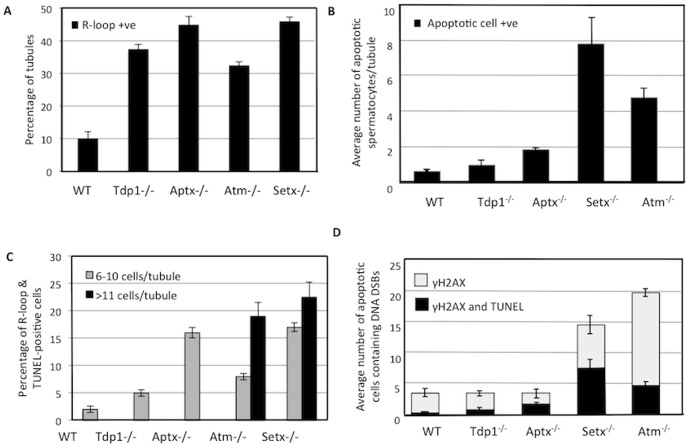
R-loop formation, apoptosis, and DNA damage quantitation in ARCAs mouse testes. **A.** Percentage of seminiferous tubules containing R-loops. **B.** Average number of apoptotic spermatocytes per tubule in testes from ARCA mice. **C.** Percentage of R-loop & TUNEL-positive cells. **D.** Average number of apoptotic cells containing DNA DSBs (γH2AX). For each panel, 200 tubules were examined per animal and cells were counted in each tubule (n = 3). Error bars represent SEM.

Since infertility is frequently associated with these disorders, we initially examined the testes for evidence of these structures. Testes sections from four ARCA mouse models (*Atm^−/−^, Setx^−/−^, Aptx^−/−^, Tdp1^−/−^*) were screened for the presence of R-loops. Initial histological examination of testes sections revealed a severe disruption of the seminiferous tubules, vacuolation and absence of mature germ cells in both *Setx^−/−^* and *Atm^−/−^* testes (Figure 1A) which is expected given that these mice have been reported to be sterile [23], [31]. On the other hand, tubules were grossly normal for both *Aptx^−/−^* and *Tdp1^−/−^*mice. Testes size was reduced in *Setx^−/−^*and *Atm^−/−^* mice but these were of normal size in the other two mutant animals (data not shown). Immunofluorescent labelling with an antibody (S9.6) specific for R-loops revealed high levels of staining in the testes sections of both *Setx^−/−^* and *Atm^−/−^* mice (Figure 1B). R-loops staining was distributed across the entire cell nucleus in agreement with previous report indicating the genome wide distribution of R-loops [25]–[28]. R-loops were also detected albeit at a lower level in both *Aptx^−/−^* and *Tdp1^−/−^* mice. To determine whether the accumulation of R-loops may impact on cell viability, co-staining with TUNEL (marker of apoptotic cells) was performed. It was observed that most cells positive for R-loops were also undergoing apoptosis (Figure 1B). Treatment of sections with RNase H prior to staining significantly reduced staining with S9.6 antibody, providing further evidence of the specificity of this antibody and that R-loops are being detected in this assay (Figure S1 A). Another characteristic of genomic instability and infertility results from a defective repair of programmed DNA DSB occurring during meiosis [23], [31]. In addition to R-loop accumulation, we also stained for γH2AX, a well-described marker for DNA DSBs [32]. DNA DSBs were detected in spermatocytes for all sections as part of the process of meiotic recombination (Figure 1C). However, the intensity of staining for γH2AX was higher in *Setx^−/−^*, *Atm^−/−^* and *Aptx^−/−^* sections. It is also evident that in the spermatocytes from *Atm^−/−^* and *Setx^−/−^*mice, the intense staining for DNA DSBs coincides with the appearance of marked TUNEL labelling (Figure 1C). These data provide evidence for the accumulation of R-loops in the presence of DNA DSBs arising as a consequence of absence of defects in proteins that are involved in the DNA damage response. Quantitative analysis demonstrated that a significantly higher number of tubules from all 4 mutant mice (∼30–45%) contained R-loops as compared to control (∼10%) (Figure 2A). This was also the case for apoptosis, with the highest levels of apoptosis observed in *Setx^−/−^* and *Atm^−/−^* mice, followed by *Aptx^−/−^* and *Tdp1^−/−^*mice (Figure 2B). It was also clear that in the case of the wildtype testes where there are only a few apoptotic cells, the great majority of these did not stain for R-loop. When tubules were differentiated based on the number of R-loop-containing spermatocytes per tubule, a clear distinction appeared. Only *Setx^−/−^* and *Atm^−/−^*mice scored in the high range with tubules containing >11 R-loop & TUNEL-positive cells per tubule (Figure 2C).Combining both high (>11 affected cells per tubule) and low (6–10 affected cells per tubule) categories, *Setx^−/−^*mice had an average of 40% R-loop-containing cells per tubule and *Atm^−/−^*mice had 30%. In the lower range, *Aptx^−/−^*mice had significantly greater numbers than wildtype mice whereas that of *Tdp1^−/−^*mice were less markedly elevated (Figure 2C). Interestingly, while *Atm^−/−^* mice showed the highest numbers of γH2AX-positive cells, only ∼25% of those stained for TUNEL (Figure 2D). This was also observed in *Tdp1^−/−^*mice (Figure 2D). In contrast, in *Setx^−/−^* and *Aptx^−/−^*, ∼50% of the cells were positive for both γH2AX and TUNEL. These data indicate that there is a higher proportion of germ cells containing DNA DSBs that undergo apoptosis in *Setx^−/−^* and *Aptx^−/−^*mice compared to *Atm^−/−^* and *Tdp1^−/−^*. The elevated levels of apoptosis in the spermatocytes of *Tdp1^−/−^*, *Aptx^−/−^*, *Setx^−/−^* and *Atm^−/−^* mice suggest that a significant amount of DNA DSBs are not processed/repaired properly, thus triggering apoptosis. As expected in wildtype, there were low levels of apoptosis which is in agreement with the formation and then effective repair of programmed DNA DSBs that occur during meiosis [33].

### R-loops do not form in post-mitotic tissues

**Figure 3:**
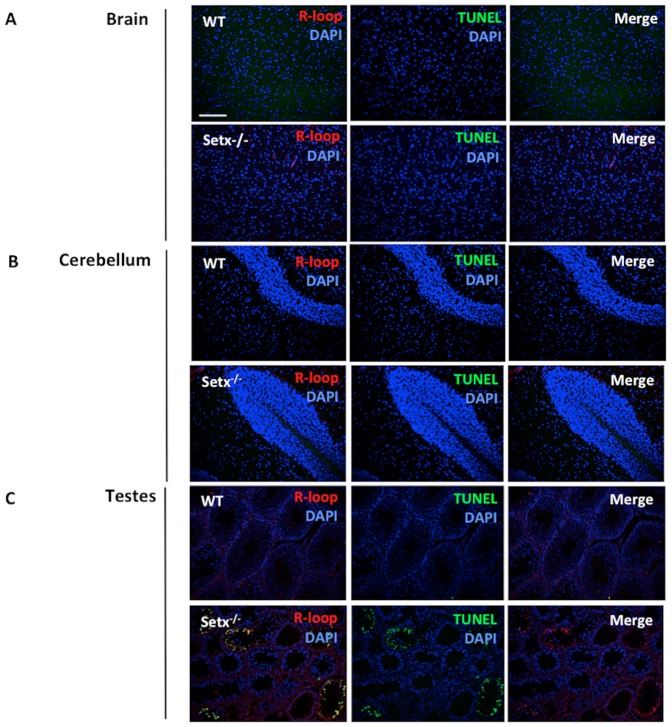
Absence of R-loops in *Setx^−/−^* nervous tissues. **A.** Whole brain sections from wildtype (*Setx^+/+^*) and *Setx^−/−^* were stained for R-loop (Red) and apoptosis (TUNEL, Green). Nuclei were labelled with DAPI. **B.** Wildtype and *Setx^−/−^* cerebellar sections staining with R-loops and TUNEL. DAPI stained nuclei. **C.** As a control for R-loop and TUNEL staining, both wildtype and *Setx^−/−^* testes sections are shown. Scale bar, 100 µm.

**Figure 4:**
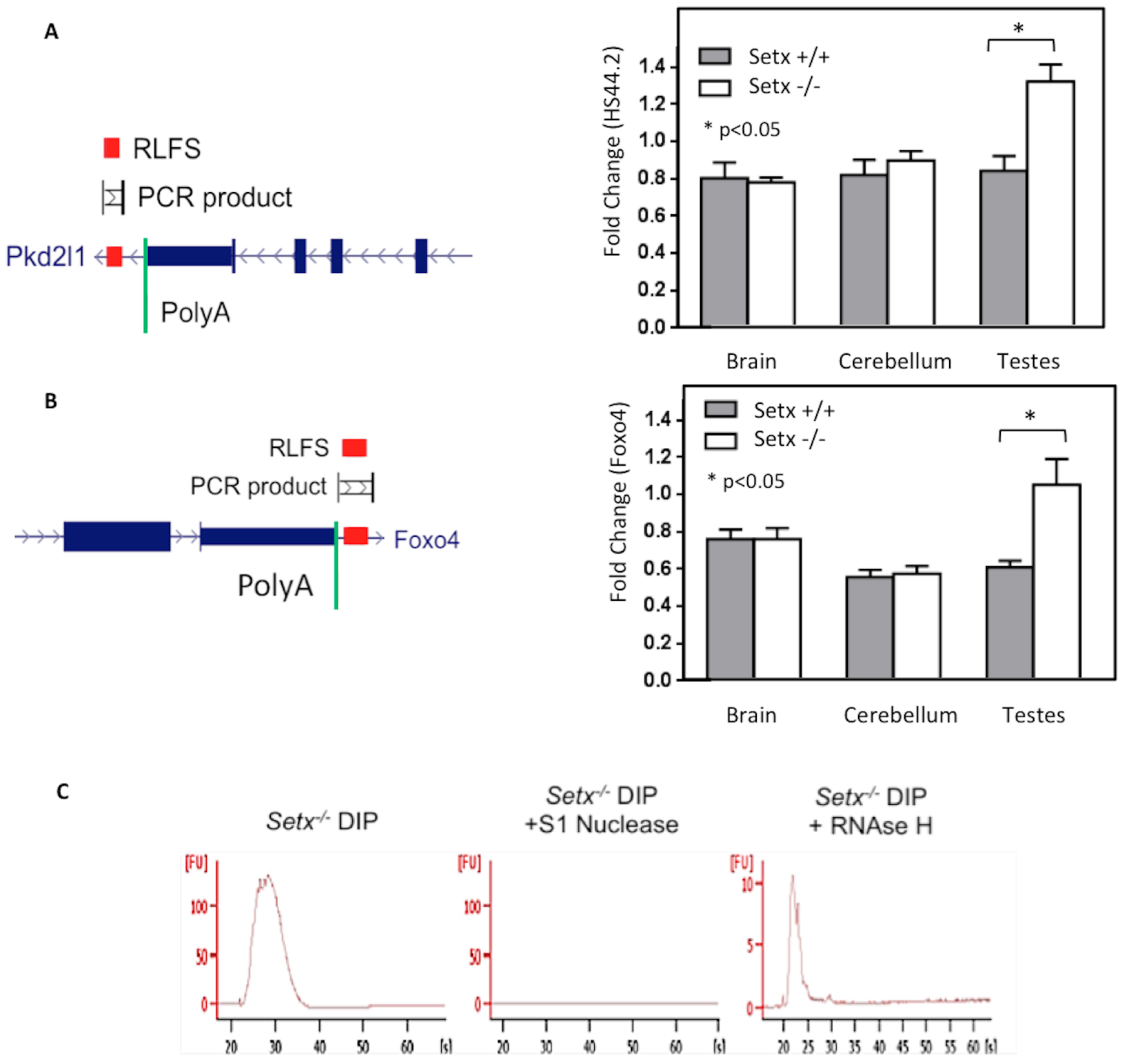
R-loops forming sequences (RLFS) prediction and R-loops detection using DRIP assay.) UCSC Genome browser shows chromosome mapping of RLFS and poly(A) signal location at 3′ UTR of studied genes. RFLS (red box), and PCR products (black vertical lines connected by horizontal lines) are indicated on the diagram. **A.** RLFS prediction in the 3′ UTR of the mouse *Pkd2l1* gene located in the HS44.2 locus on chromosome 19. See Figure S5 for sequence details. DRIP quantitation from *Setx^+/+^* and *Setx^−/−^* showed similar background levels of R-loops in brain and cerebellum. Only a significant increase in R-loop formation was observed in *Setx^−/−^*testes as compared to *Setx^+/+^*(Students t-test, p<0.05, n = 3). **B**. RLFS prediction in the 3′UTR of the mouse *Foxo4* gene located on the X chromosome. DRIP quantitation from *Setx^+/+^* and *Setx^−/−^* showed similar background levels of R-loops in brain and cerebellum. Similarly, a significant increase in R-loop formation was only observed in *Setx^−/−^*testes (Students t-test, p<0.05, n = 3). **C.** Treatment of *Setx^−/−^* DRIP samples with S1 Nuclease and RNAse H confirmed the specificity of the S9.6 (R-loop) antibody towards DNA/RNA hybrids as shown by reduced fluorescence intensities on Bioanalyzer Spectra.

To date R-loops have been detected in cells/tissues where active transcription and replication occur [34], [35]. However, the most debilitating aspect of autosomal recessive cerebellar ataxias is caused by the progressive loss of post-mitotic nerve cells, leading to cerebellar atrophy and peripheral neuropathy [5]. Post-mitotic neurons are metabolically highly active due to the synthesis of neurotransmitters to ensure proper function of the nervous system and therefore possess a high transcriptional activity [36]. Given that R-loops form normally during transcription at RNA Polymerase II pause sites and that post-mitotic neurons display high levels of transcription and RNA processing, it raises the question as to whether R-loops form or accumulate in the nervous tissues of disorders characterized by the defects in proteins involved in the DNA damage response. Given the role of senataxin in resolving these structures [24], we initially examined brain sections from *Setx^−/−^* mice for the presence of R-loops. However, we did not detect any R-loops in either whole brain or cerebellum sections from *Setx^−/−^*mice (Figure 3A & 3B). Furthermore, there was also no evidence of cells undergoing apoptosis in these tissues (Figure 3A & 3B). This is in agreement with the fact that no neurological anomalies or neurodegeneration were present in these animals [23]. Testes sections from *Setx^−/−^* animals were included as positive control for R-loop staining (Figure 3C). Accumulation of R-loops observed in *Setx^−/−^* germ cells and their absence in the brain and cerebella was supported using DRIP (DNA/RNA immunoprecipitation) assays for *Setx^+/+^* and *Setx^−/−^* testes, brain, and cerebella. In order to perform DRIP assays, we first searched for R-loop prediction using the R-loop forming sequence (RLFS) model to predict location of RLFSs on the mouse genome [37]. Given the role of R-loops in transcription termination [24], we selected candidate genes where RLFSs localized <500 bp downstream of the poly(A) signal(s) for further analyses. Among these genes, the HS44.2 locus, a region of recombination hot spot located on the chromosome 19 [38] was selected. The RLFS-containing region was located downstream the *Pkd2l1*gene located in the HS44.2 locus (Figure 4A). We observed similar background levels of R-loop formation in both *Setx^+/+^* and *Setx^−/−^* brain and cerebellum. However, a significant increase (>1.6-fold) in R-loop formation was observed in *Setx^−/−^* testes compared to wildtype supporting our immunofluorescence findings (Figure 2). In addition, given the meiotic sex chromosome inactivation (MSCI) defect previously observed in *Setx^−/−^* [23] we selected a second locus on the X chromosome to perform the DRIP analysis. We selected the *Foxo4* gene based on the same criteria. Similar results were obtained for *Foxo4*, confirming background levels of R-loops in brain and cerebellum and a significant increase of R-loop formation (>1.8 fold) in *Setx^−/−^* testes (Figure 4B). Figure 4C shows the Bioanalyzer Spectra of untreated, S1 Nuclease-treated, and RNAse H-treated DRIP samples to confirm the specificity of the S9.6 antibody (R-loop) for DNA/RNA hybrids as shown by the reduction of fluorescence intensity after RNAse H treatment. Altogether, these data confirmed the specific accumulation of R-loops in *Setx^−/−^* germ cells and the presence similar normal background levels of R-loops in *Setx^+/+^* and *Setx^−/−^* brain and cerebellum, in agreement with the fact that no neurological anomalies or neurodegeneration were present in these animals [23]. Thus, S9.6 immunofluorescence staining represents a simple and reliable method to detect R-loop formation and screen for aberrant RNA metabolism in tissues.

To determine whether this was also the case in other ARCA models, we screened for R-loop accumulation in the brains and cerebella of *Atm^−/−^*, *Aptx^−/−^* and *Tdp1^−/−^* mice. Similar to that in *Setx^−/−^*, we did not detect these structures in these tissues (Figure S1 A and B). The lack of R-loops in the nervous tissues of these autosomal recessive cerebellar ataxias may not be entirely unexpected due to the lack of major neurological defects in these animals.

### DNA damage induces R-loops accumulation in proliferating cells not in post-mitotic cells

**Figure 5:**
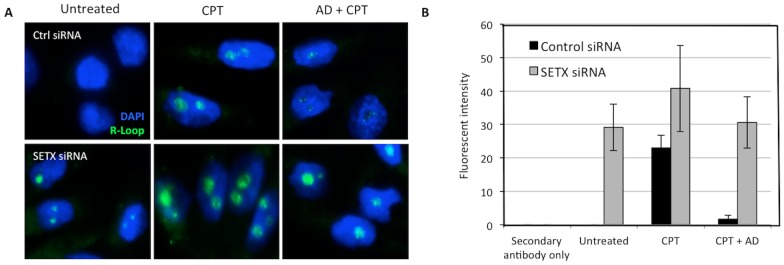
Induction of R-loop after DNA damage exposure. **A.** Knock down of senataxin in HeLa cells using short interfering RNA (siRNA) induced the accumulation of R-loops in nucleoli. Knock down efficiency for senataxin is shown in Figure S2. Treatment of these cells with 25 µM of camptothecin (CPT) led to the accumulation of R-loops in control cells (Ctrl siRNA) and a further increase in senataxin knockdown cells (*SETX* siRNA). Pre-treatment of these with RNA polymerase inhibitor Actinomycin D (AD, 5 µg/ml), ablated the CPT-induced formation of R-loop in both cell types. **B.** Quantitation of R-loops formation in control and senataxin knockdown cells. No R-loops were detected in ctrl siRNA-treated cells under normal growing conditions. CPT induced the formation of R-loops in both cell types and pre-treatment these cells with AD prevented the induction of R-loops following DNA damage exposure. Levels of R-loops returned to basal levels in *SETX* siRNA-treated cells after addition of AD.

The accumulation of unrepaired DNA damage in post-mitotic neurons can lead to genome instability, abnormal transcriptional regulation and thus ultimately to neurodegeneration [30]. The lack of R-loop formation in the nervous tissues of these mutants under normal living conditions prompted us to investigate whether additional DNA damage exposure may trigger the formation of these structures. To address this, we first knocked down *SETX* in HeLa cells (Figure S2) and monitored R-loop formation in these cells. As shown in Figure 5A, R-loops were primarily detected in nucleoli. This is compatible with R-loop formation at highly transcribed and R-loop-prone ribosomal arrays as previously reported [39]. The major nucleolar R-loops staining observed here is consistent with the staining pattern found in previous studies that reported the genome wide distribution of R-loops using DRIP assays [40]. Ribosomal DNA transcription represents the vast majority of transcriptional activity in the cell thus explaining the strong R-loop signal observed in nucleoli. The detection of R-loops in nucleoli does not mean that R-loops only form in nucleoli but may reflect a difference in sensitivity between immunofluorescence and DRIP assays. As shown in Figure 4, we detected the formation of R-loops at non-rDNA loci on two different chromosomes using DRIP assays thus confirming that R-loops occur on a genome wide scale. Extra-nuclear staining for R-loops was also observed in agreement with the formation of R-loops during mitochondrial replication as previously reported [41], [42]. These data confirm the role for senataxin in resolving R-loops as previously described [21], [23], [24]. Exposure of cells to the DNA damaging agent, camptothecin (CPT), a DNA topoisomerase I inhibitor, led to the appearance of R-loops in nucleoli in control cells and a further increased levels of R-loops in SETX knockdown cells, in keeping with the higher basal level in these cells (Figure 5A). The accumulation of positive supercoiling ahead of the transcription bubble resists opening of the DNA, which can slow or impede transcription elongation by RNA Polymerase I [43], as seen following treatment with CPT [44]. In contrast, negative supercoiling behind the transcription bubble can lead to the unwinding of DNA. When this happens, the nascent RNA may hybridize to the transcribed strand, creating R-loops [44]. The additional increase in R-loop formation observed after CPT treatment supports previous findings, showing that the blocking of DNA topoisomerase I leads to R-loop-mediated transcriptional stalling during ribosomal RNA synthesis [39], [40]. To confirm that R-loop formation requires active transcription, we employed the use of Actinomycin D (AD), a transcription inhibitor that binds DNA at the transcription initiation complex and prevents elongation of RNA by RNA polymerases [45]. Following AD treatment, R-loop levels decreased in both control siRNA- and *SETX* siRNA-treated cells (Figure 5A and 5B), confirming the involvement of active transcription in R-loop formation.

**Figure 6:**
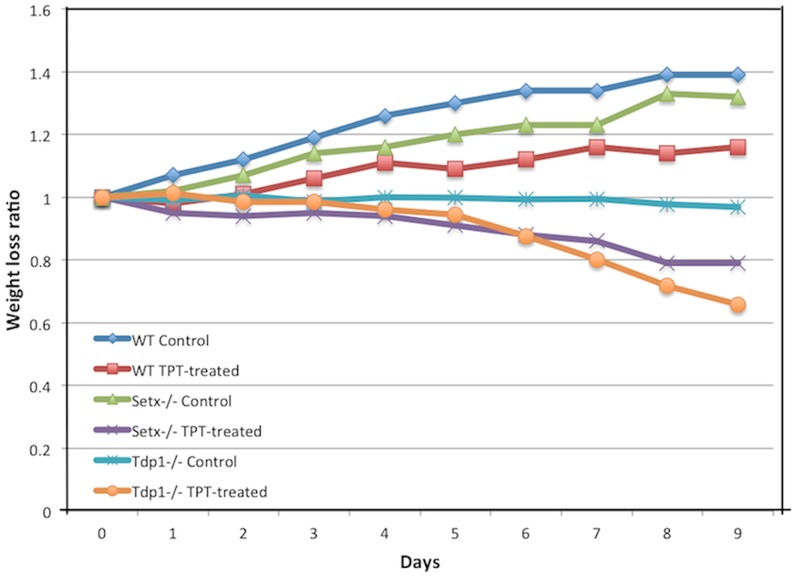
Treatment of *Setx* and *Tdp1* mice with Topotecan induces severe weight loss. Wiltype (WT), *Setx^−/−^* and *Tdp1^−/−^* mice were treated with a daily dose of topotecan (TPT, 2 mg/kg/day) over a period of 9 days in order to exacerbate the formation of R-loops in these mice. Mice of each genotype were injected daily with a dose of TPT (2 mg/kg/day) and weights were recorded daily (n = 3). Controls were injected with an equivalent volume of purified water.

**Figure 7:**
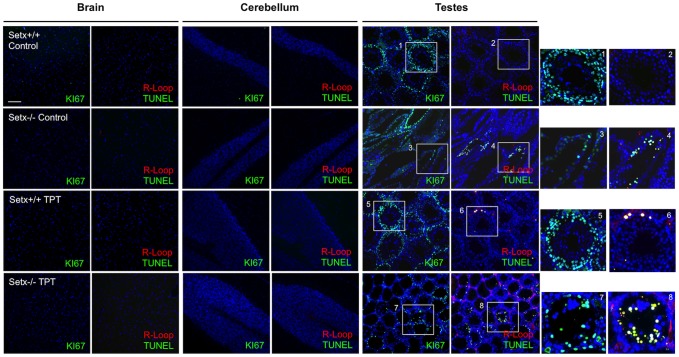
Topotecan treatment does not induce R-loops formation in post-mitotic nervous tissues in *Setx^−/−^* mice. Histological sections of brain, cerebellum, and testes were immunostained for R-loops (Red) and TUNEL (Green). No R-loops were detected in brain and cerebellum sections after TPT treatment. In contrast, an increase in the number of R-loop-containing cells was observed in wildtype and *Setx^−/−^* after TPT exposure suggesting that R-loop preferentially form in proliferating cells, most likely due to collision between the DNA replication machinery and the transcription apparatus. DAPI stained nuclei and Ki67 (Red) was used as a marker for proliferation. Scale bar, 100 µm.

**Figure 8:**
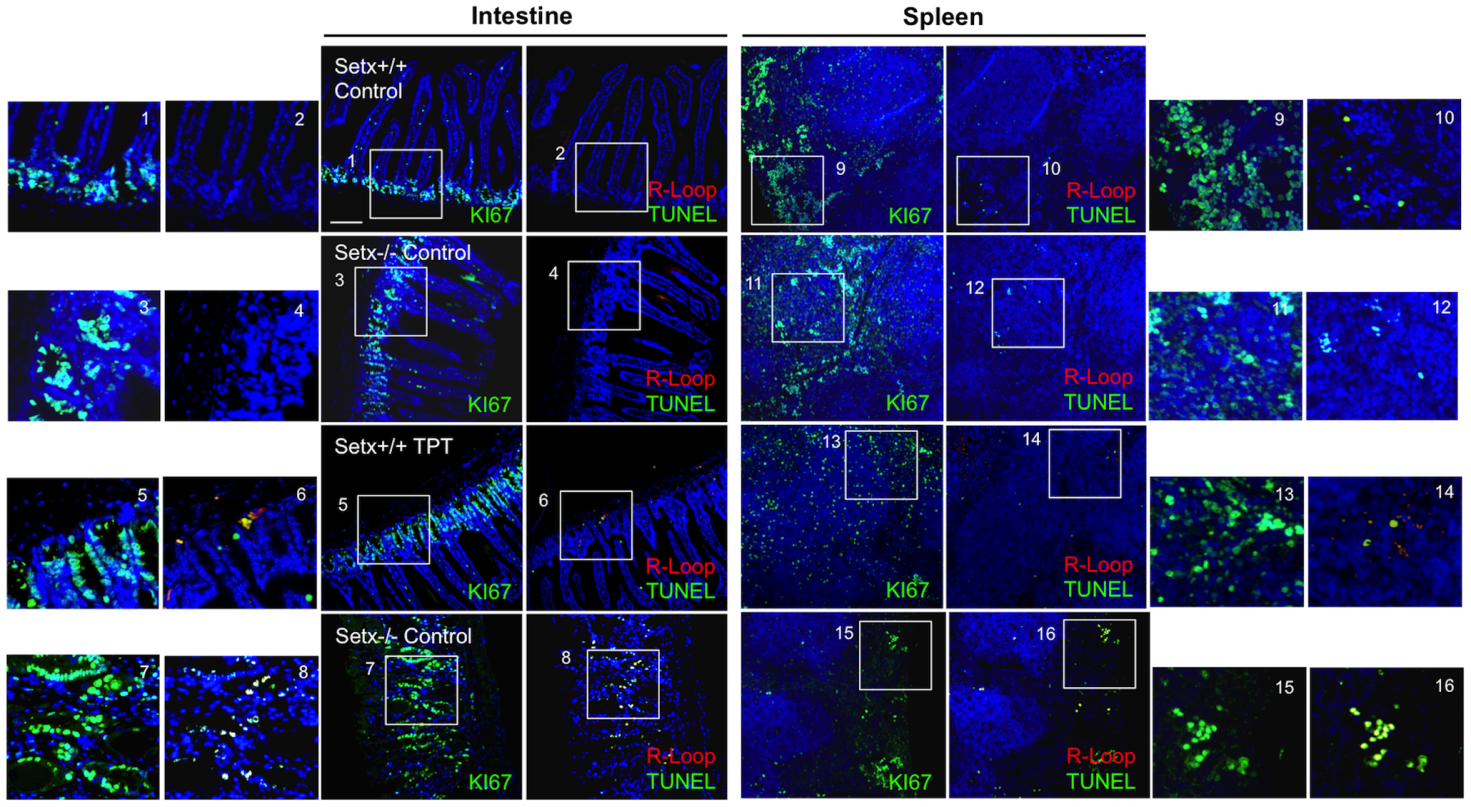
Induction of R-loops in proliferating cells after topotecan treatment. Wildtype and *Setx^−/−^*histological sections of intestine and spleen, two tissues with a high proliferative capacity, were stained for R-loops, TUNEL, and Ki67. TPT induced the formation of R-loops and apoptosis in both intestine and spleen. TPT disrupted the structure of the small intestine in *Setx^−/−^* animals supporting the severe weight loss observed in these mice. R-loop (red), TUNEL (green) and Ki67 (red). DAPI (blue) stained nuclei. Scale bar, 100 µm.

**Figure 9:**
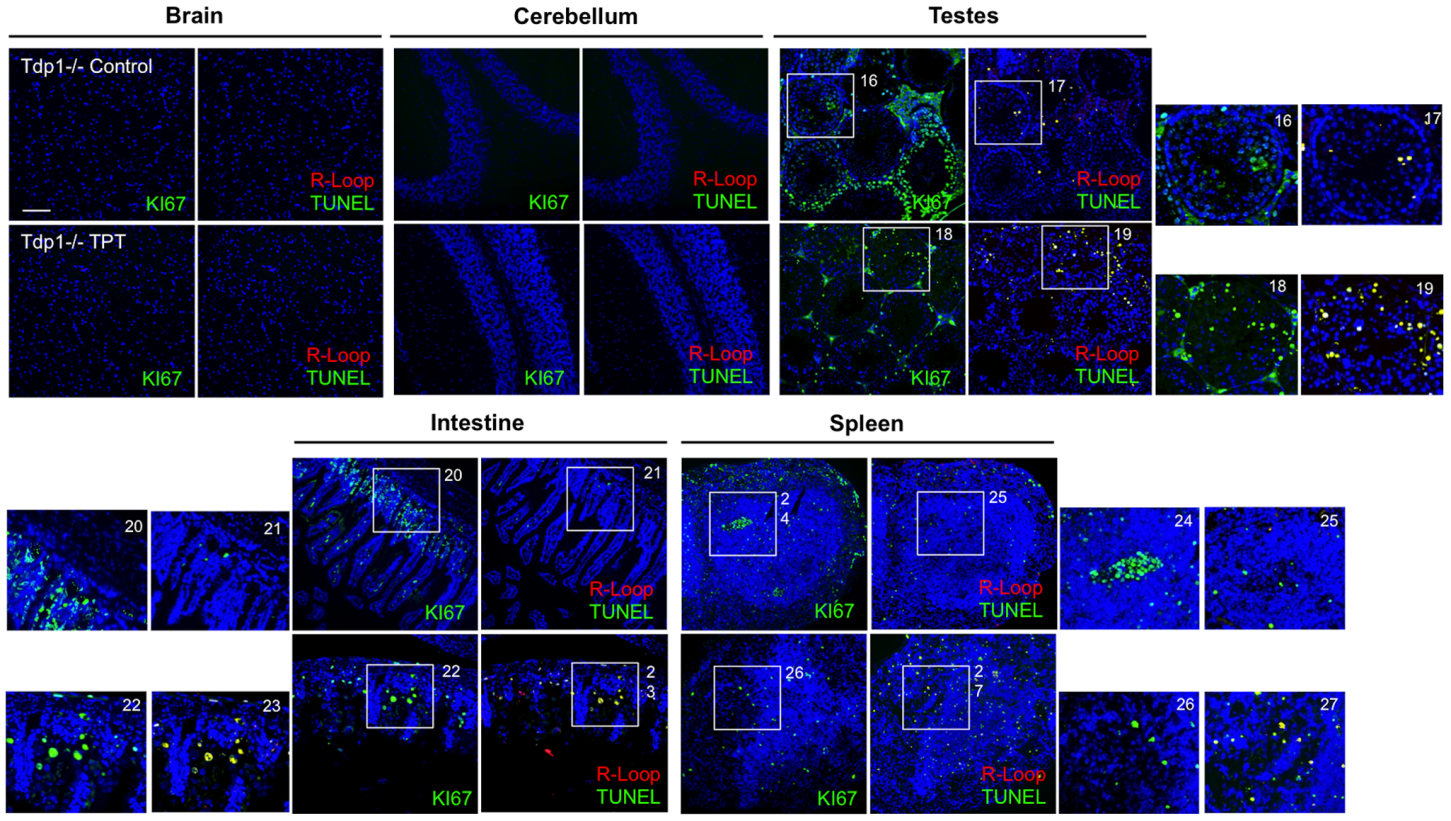
Induction of R-loops only in proliferative tissues in *Tdp1^−/−^* after topotecan treatment. Histological sections from *Tdp1^−/−^* mice were stained for R-loops, TUNEL and Ki67 after topotecan exposure. Similar to *Setx^−/−^*, TPT induced the formation of R-loops formation only in proliferative tissues testes, intestine and spleen). No R-loops were detected in post-mitotic tissues (brain and cerebellum). Scale bar, 100 µm.

**Figure 10:**
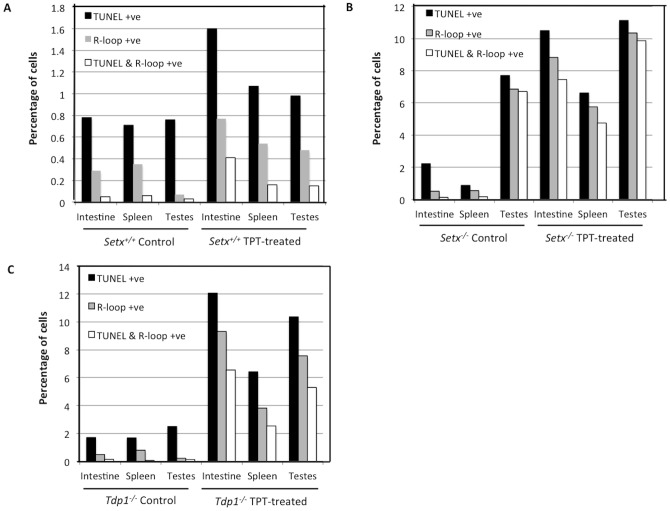
Increased R-loop formation in TPT-treated knockout *Setx* and *Tdp1* mice. Graphs show the percentage of apoptotic (TUNEL) cells only, cells containing R-loops only and apoptotic cells containing R-loops in the different tissues of *Setx* (**A** and **B**) and *Tdp1* mice (**C**). Counts were performed using 5 fields of view per tissue per animal (n = 3). Higher levels of these three cell types were observed in TPT-treated knockout animals.

Having established that CPT-induced DNA damage can stimulate R-loop formation; we then treated wildtype, *Setx^−/−^* and *Tdp1^−/−^* mutant mice with topotecan (TPT), a water-soluble analogue of CPT [46]. Knockout mice of both models treated with this agent lost approximately 30% of body weight over a 10-day period (Figure 6). This may have resulted from damage to the mucosal tissue in the lower intestine, causing disruption to nutrient absorption. It has also been shown that TPT can cause gastrointestinal toxicity in mice [18], [47]. However, no abnormal neurological behaviour was observed in TPT-treated animals as compared to untreated controls over the treatment period. We then screened for the presence of R-loops in both proliferating and post-mitotic tissues of these mice using the R-loop-specific S9.6 antibody together with the Ki67 antibody, which was used as a marker of proliferation. As expected, in the testes of *Setx^−/−^* mice, coinciding R-loop and Ki67 signals were detected under untreated conditions [23] and this increased after TPT treatment (Figure 7). However, examination of the brain and cerebella tissues from both treated and untreated *Setx^−/−^* mice failed to reveal evidence of R-loop formation (Figure 7). Although TPT is able to readily cross the blood-brain barrier and has been demonstrated to be active in brain metastases from small cell lung cancer [48], it did not induce the formation of R-loops in these animals. Since increased R-loop levels were only observed in a proliferative tissue such as the testes, we then investigated for the presence of R-loops in other highly proliferative tissues such as the intestine and the spleen. Increased levels of coinciding R-loop and Ki67 signals were detected in these tissues after TPT treatment (Figure 8). Similar results were found in *Tdp1^−/−^* mice (Figure 9). Quantitation of the number of R-loop-, TUNEL- and R-loop & TUNEL-positive cells is shown in Figure 10. Only a very small number of R-loop positive cells (<0.4% of cells) were detected in *Setx^+/+^* intestine, spleen and testes. Treatment of *Setx^+/+^* mice with TPT lead to a 2.7-fold, 1.5-fold, and 6.8-fold increase in the number of R-loop formation cells in intestine, spleen and testes, respectively (Figure 10A). As anticipated, *Setx^−/−^* mice exhibited higher levels of R-loop formation in intestine, spleen and testes, with testes showing the highest number of R-loop-positive cells (6.9% of cells). Following TPT treatment, the number of R-loops-containing cells in *Setx^−/−^* mice drastically increased in all three tissues by a 17.3-fold in the intestine, 10.5-fold in the spleen, and 1.5-fold in the testes which remained the tissue containing the highest number of R-loops-positive cells (Figure 10B). Similar results were observed for *Tdp1^−/−^*mice (Figure 10C). Less than 0.5% of R-loops-positive cells were detected in *Tdp1^−/−^* mice under normal conditions. TPT treatment of *Tdp1^−/−^* mice lead to a drastic increase in the number of R-loops-positive cells with intestine and testes displaying a 19-fold and 34.4-fold increase, respectively (Figure 10C). As a consequence of the TPT-induced R-loop accumulation, the number of double-positive cells (R-loop & TUNEL) after TPT treatment dramatically increased in *Setx^+/+^*, *Setx^−/−^* and *Tdp1^−/−^* mice further supporting the effect of R-loop accumulation in genome stability. We next examined the levels of senataxin protein in these tissues using immunoblotting. We failed to detect senataxin protein using western blotting of wildtype total tissue extracts, suggesting very low levels of senataxin in these tissues and/or weak sensitivity of the senataxin antibody. We thus performed senataxin immunoprecipitation from wildtype and *Setx^−/−^* tissues extracts. As seen in Figure S4 A, a weak signal for senataxin was only detected in *Setx^+/+^* testes extracts. This is agreement with the GEO expression data, which showed the highest expression for senataxin in testes as compared to other tissues. Using RT-PCR, we confirmed the low levels of senataxin in brain, cerebellum, spleen and intestine compared to testes (Figure S4 B).

Altogether, these data indicate that R-loops form preferentially in replicating cells of proliferative tissues due to the collision of the replication fork and the transcription machinery [21], [27], and not in post-mitotic cells such as the neurons in the brain and cerebellum even after DNA damage exposure.

Given that the loss of TDP1 leads to an age-dependant and progressive cerebellar atrophy, and that cerebellar neurons or primary astrocytes derived from *Tdp1^−/−^* mice display an inability to rapidly repair DNA SSBs associated with Top1-DNA complexes or oxidative damage [18], we generated *Setx^−/−^Tdp1^−/−^* double mutants to investigate whether the compounded defects would induce or exacerbate R-loop formation and trigger a neurological phenotype. However, while male sterility was observed in the double knockout, no neurological defects were observed. Similar results to the single *Setx^−/−^* and *Tdp1^−/−^* knockout TPT-treated mice were observed in the double knockout, with similar levels of R-loop accumulation seen in the testes, spleen and intestine but not in the brain or cerebellum (data not shown).

## Discussion

Recent evidence suggests that senataxin plays a central role at the interface between transcription, DNA replication and genetic recombination in resolving R-loops to minimise the risk of genome instability [21], [23], [24]. In this report, we demonstrate that the disruption of a number of DNA damage response genes including *Setx* and *Tdp1* in mice led to the accumulation of R-loops in proliferating cells consistent with collision between transcription and DNA replication forks when DNA lesions were not repaired [21], [27]. However, there was no evidence of the accumulation of R-loops in the nervous tissues of these gene-disrupted animals even when DNA damage was inflicted. These data suggest that in post-mitotic cells, where transcription is highly active, only basal levels of R-loops exist and they do not accumulate even in the presence of DNA damage. Thus this is a phenomenon that appears to be restricted to proliferating cells. We have previously shown that the disruption of *Setx* in mice caused persistence of DNA DSB, a defect in Rad51 disassembly, accumulation of R-loops and a failure of crossing-over in spermatocytes [23]. Here, we showed that R-loops accumulate in the testes even when other DNA damage response genes were disrupted. Mutations in the ATM gene give rise to ataxia-telangiectasia (A-T), which is characterised by immunodeficiency, cancer predisposition, neurodegeneration and also infertility [5]. *Atm^−/−^* spermatocytes halt development largely at leptotene stage of meiotic prophase 1 and ovaries from females contain no primary oocytes or follicles [49]. The integrity of axial elements and synaptonemal complexes were disrupted and DNA DSB persisted in *Atm^−/−^*spermatocytes consistent with a role for ATM in monitoring DNA DSB during meiotic recombination in early leptotene. ATM responds to the presence of excess breaks by inducing apoptosis. In this study we detected excess DNA DSB in testes sections from *Atm^−/−^*mice and these co-localised with R-loops suggesting that a combination of blocked transcription together with unrepaired DNA DSBs is responsible for the disrupted spermatogenesis. It is also evident that the majority of R-loop-containing spermatocytes are undergoing apoptosis. Mutations in *TDP1* give rise to spinocerebellar ataxia with axonal neuropathy (SCAN1) [17]. Mice disrupted for this gene display an inability to rapidly repair SSB [18]. There is also a requirement for this enzyme in the removal of acutely elevated levels of Topo1-associated DNA strand breaks in intestinal and haematopoietic progenitor cells. Although *Tdp1^−/−^*mice are fertile and do not exhibit the same high level of R-loop accumulation and cell death in testes sections as *Setx^−/−^* and *Atm^−/−^* mice, we did detect levels of R-loops that were significantly higher than wildtype and there was concordance between these and apoptosis. This suggests that there is elevation in DNA breaks in *Tdp1^−/−^*mouse spermatocytes or increased DNA replication blockage that leads to R-loop accumulation. Mutations in the *APTX* gene lead to ataxia oculomotor apraxia type 1 (AOA1). *APTX* codes for aprataxin, which is an enzyme that catalyzes the removal of abortive DNA ligation intermediates and is important for DNA SSB repair [15], [16]. The fourth mutant mouse model, *Aptx^−/−^* which also exhibits normal fertility, had elevated numbers of R-loops in the testes as compared to wildtype and similar to the *Tdp1^−/−^*mouse. However it was not to the same extent as that seen in the *Setx^−/−^* and *Atm^−/−^*mice. These data indicate that in the absence of proteins involved in recognition and/or repair of DNA damage, R-loops occur in proliferating cells due the presence of unrepaired DNA lesion that can stall the transcription machinery. In addition, the extent of R-loop accumulation can exacerbate genomic instability, trigger apoptosis and is indicative of the fertility status.

**Figure 11:**
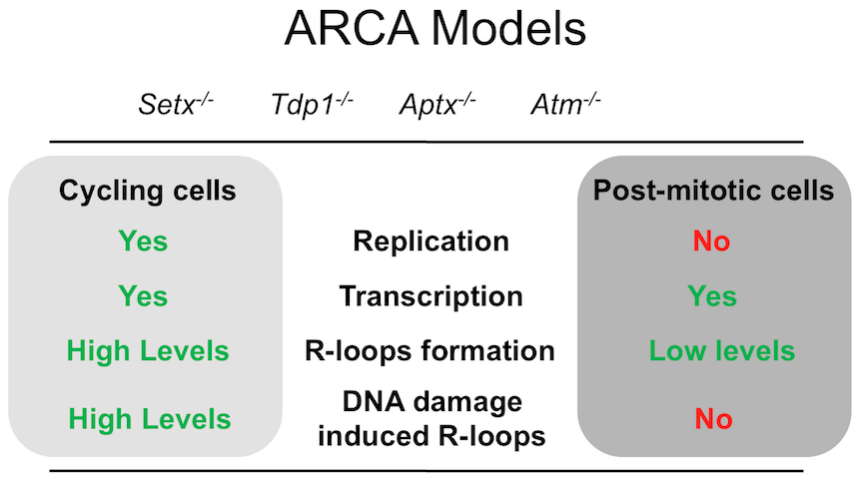
R-loops preferentially form in cycling but not in post-mitotic cells. Model depicting the requirements for R-loops formation in the various ARCAs model. While transcription is implied in the formation of R-loops, active replication appears to be a necessary condition too. Thus R-loops accumulation does not appear to contribute to the neurodegenerative phenotype observed in autosomal recessive cerebellar ataxias.

All four mutants studied here correspond to autosomal recessive ataxias characterised by cerebellar atrophy and loss of Purkinje cells [50]. While the syndromes vary in the extent of extra-neuronal involvement, they are all characterised by neurodegeneration. Although the mouse models for the disorders described here do not recapitulate the neurodegeneration seen in human patients, some neurological abnormalities can be observed. For example, in the case of *Tdp1^−/−^*mice, there is an accumulation of Topo1-linked breaks in astrocytes and an age-dependent reduction in cerebellar size [18]. *Atm^−/−^*mice also exhibit cerebellar pathology, neuronal cell death *in vitro*, reduced synchronization persistence in neural networks, vascular abnormalities and degeneration of the nigro-striated pathway [51]. Neuronal cells are characterised by active transcription and high levels of metabolic activity [36]. As such, it seemed likely that in the presence of unrepaired DNA breaks, R-loops would accumulate in the brains of these mutant mice. Only background levels of R-loops were detected in either the brain as a whole or cerebellum using the more the sensitive DRIP assay. However, treatment of any of these animals with TPT, which is a compound that readily crosses the blood-brain barrier and induces DNA damage, did not trigger accumulation of R-loops in post-mitotic tissues. This suggests that while R-loops occur normally at pause sites during transcription [52], their accumulation is only observed in cells where transcription collides with either DNA replication forks [21] or with DNA recombination intermediates during meiosis (Figure 11) [23]. Clearly neither of these events occur in post-mitotic neurons. While R-loops have been identified as a substrate for senataxin [24], it is possible there are other substrates which might be more important in post-mitotic cells. In this context, it has been shown that senataxin functions at different stages during transcription [22]. For instance, the binding of RNA polymerase II to candidate genes was significantly reduced in senataxin deficient cells and consequently the expression of these genes. Transcription termination was also abnormal as determined by transcript readthrough [22]. This was demonstrated in senataxin-depleted cells where an over-expression of RNAse H resolved R-loops and caused transcriptional readthrough [24]. Suraweera et al 2009 also demonstrated that the splicing efficiency of specific mRNAs and alternate splice site selection were altered in senataxin-deficient cells [22]. The absence of R-loops in post-mitotic tissues may reflect a regulatory role for senataxin in transcription and/or RNA processing that does not involve the resolution of R-loops *per se*. Vantaggiato et al. 2011 reported a role for senataxin in neuronal differentiation through fibroblast growth factor 8 (FGF8) signalling where they found that an overexpression of senataxin was sufficient to trigger neuronal differentiation in primary hippocampal neurons or in P19 cells treated with retinoic acid [53]. FGF8 is one of several growth factors that regulate neurogenesis, neuronal differentiation, survival and synaptic plasticity, both during development and in adulthood [53].

Cellular viability depends on the well-orchestrated functions carried out by numerous protein-coding and non-coding RNAs, as well as RNA-binding proteins. Mutations or abnormalities that disrupt RNA or protein components of RNP complexes can be deleterious to cells and cause disease states. During the last decade, it has become increasingly evident that abnormalities in RNA processing represent a common feature among many neurodegenerative diseases. For instance, some neurodegenerative diseases result from mutated coding and non-coding RNAs, and misregulation of long non-coding RNA transcription [54], [55]. “RNAopathies” include diseases caused by non-coding repeat expansions and RNAs that exert toxicity via diverse mechanisms such as RNA foci formation, bidirectional transcription, and the production of toxic RNAs and proteins by repeat associated non-ATG translation [29]. Much work remains in order to elucidate the mechanisms underlying “RNA-binding proteinopathies”, such as amyotrophic lateral sclerosis (ALS) in which the RNA-binding protein TDP-43 plays a prominent role [54]. The absence of R-loops formation in post-mitotic neuronal cells as observed here in this study suggests that AOA2 may represent a novel “RNAopathy” in which defects in senataxin can affect the fidelity of the transcriptome. Further investigation of transcriptional regulation, RNA processing, and gene expression in AOA2 awaits the generation of an appropriate neuronal model for this disease.

## Materials and Methods

### Ethics Statement

All animal work and experiments have been approved by The QIMR Berghofer Medical Research Institute Animal Ethics Committee and all efforts were made to minimize suffering.

### Animal housing, husbandry and genotyping

The *Setx* (C57Bl6/129Sv), *Tdp1* (C57Bl6), *Aptx* (C57Bl6) and *Atm* (C57Bl6/129Sv) mouse strains were bred in house (QIMR Berghofer Medical Research Institute Animal House) and have previously been characterised and described [18], [23], [56], [57]. Heterozygote mice for *Setx^+/−^* and *Atm^+/−^* were crossed to generate homozygote knockouts since both knockouts animals are sterile. *Tdp1^−/−^* and *Aptx^−/−^* were used for breeding since these animals are fertile. The mice were housed in a specific pathogen Free (SPF) PC2 facility in OptiMICE caging system (Opti-Polycarbonate cages) with artificial bedding material and subjected to a light/dark cycle of 12 h/12 h. Temperature of the animal facility was maintained constant (21°C–23°C) and the mice had permanent access to food and water (24 h/7days). Mice were housed with 2–5 companions per cage and had an enriched environment composed of shredded paper and toilet rolls. Welfare-related assessments were carried out prior to, during and after the experiment to ensure minimal suffering. The mice were ear clipped for identification at 10 days post-partum, genotyped, and weaned at 21 days post-partum. Genotyping was carried out by PCR on genomic DNA isolated from tail tips. Genotyping for the *Setx*, *Tdp1*, *Aptx* and *Atm* mice has been previously described [18], [23], [56], [57]. Primer pairs are detailed in Figure S3. All experiments were carried out on adult (1 month-old) male animals. Tissue collection was performed after euthanasia (CO_2_ gas) as instructed by Animal welfare guidelines. Animal colonies were regularly screened every 3 months for health and immune status.

### Histological analysis of ARCAs mice testes

Testes from adult (1 month-old) male mice were collected and fixed in PBS buffered 10% formalin, embedded in paraffin block and sectioned at 4 µm. Sections were stained with Hematoxylin and Eosin (H&E). Slides were examined under light microscope and then scanned using Scanscope CS system (Aperio Technologies, Vista, USA). Images corresponding to ×10 and ×20 magnification were captured and assembled into Adobe Photoshop 7 (Adobe Systems Inc, USA).

### TUNEL assay for apoptosis

Paraffin sections from testes, brain, cerebellum, spleen and intestines were dewaxed and rehydrated with Shandon Varistain Gemini ES (Thermo Scientific, USA). Apoptotic cells were detected by Terminal deoxynucleotidyl transferased UTP Nick End Labelling (TUNEL) assay using the Fluorescence *in situ* Cell Death Detection Kit (Roche, Switzerland) following the manufacturer's instructions. TUNEL is a method for detecting DNA fragmentation by labelling the terminal end of nucleic acids and a common method for detecting DNA fragmentation that results from apoptotic signalling cascades. DNA was stained with DAPI (1∶10,000; Sigma-Aldrich) for 10 min at room temperature, and slides were mounted in Celvol 603 medium. Images were captured at room temperature using a digital camera (AxioCamMrm, Carl Zeiss Microimaging Inc., Germany) attached to a fluorescent microscope (Axioskop 2 mot plus, Carl Zeiss Microimaging Inc., Germany) and the AxioVision 4.8 software (Carl Zeiss, Microimaging Inc. Germany). The objectives employed were a Zeiss Plan Neofluar ×10/0.30 (mt10 magnification) or a 63× Zeiss Plan Apochromat 1,4 Oil DIC (Carl Zeiss, Germany). Images were subsequently assembled in Adobe Photoshop 7 (Adobe Systems Inc, USA), and contrast and brightness were adjusted on the whole image panel at the same time. For double staining, TUNEL was carried out first followed by immunostaining as described below.

### R-loop and DNA damage immunostaining on tissue sections

Slides with tissue sections were dewaxed and enzymatic antigen retrieval was performed by incubating the sections with 1∶10 Trypsin dilution in PBS for 20 min at 37°C. Slides were washed 3 times for 5 min with PBS at room temperature for 5 min each. Tissues sections were blocked in (20% FCS, 2% BSA, 0.2% Triton X-100) for 1 h at room temperature. Slides were incubated with anti-R-loop (1∶100, S9.6), anti-γH2AX (1∶100, Y-P1016, Millipore) or anti-Ki67 (1∶100, ab15580, Abcam) antibodies overnight at 4°C in a humidified chamber. Slides were washed 5 times with 1× PBS containing 0.5% Triton X-100 for 5 min each at room temperature. Alexa-Dye488 or Alexa-Dye594-conjugated secondary antibody (Molecular Probes, Life technologies) was added for 1 h at 37°C in a humidified chamber. Subsequently, slides were washed 3 times as before and DAPI (1∶10,000; Sigma-Aldrich) was added for 10 min to staining nuclei. Slides were finally washed twice and glass coverslips were mounted in Celvol 603 medium for imaging. Imaging was performed as described above. Confirmation of R-loop specific staining was obtained by pre-treating *Setx^−/−^* testes sections with RNAse H (New England Biolabs, USA) (Figure S1 A).

### siRNA transfection into HeLa cells and CPT treatment

HeLa cells (ATCC) were transfected with Stealth RNAi Negative Control LO GC (#12935200) (Invitrogen) and Stealth RNAi specific for the knock-down of *SETX* (Invitrogen): *SETX* 1 (CCAUCUAACUCUGUACAACUUGCUU) and *SETX* 3 (CCAAUUGCUCCUUUCAGGUGUUUGA). Approximately 2.5–10×10^5^ cells were transiently transfected in a six-well plate with control/*SETX* RNAi oligoribonucleotide (5 µl of a 20 µM stock) using Lipofectamine 2000 (Invitrogen) as described by the manufacturer. Knock-down efficiency of *SETX* was determined 48 h post-transfection by immunoblotting of whole cell extracts and immunostaining using and anti-senataxin (1∶2000, Ab-1) antibody [20]. PARP-1 protein levels (1∶1000, MCA1522G, Serotec) were used as the loading control. Control and *SETX* siRNA-tranfected cells were treated with 25 µM of camptothecin (CPT, Sigma-Aldrich) for 2 hours at 37°C/5% CO2 to induced DNA damage and then fixed with 4% PFA and processed for immunostaining as previously described to detect R-loop formation [20], [23]. Control and *SETX* siRNA-transfected cells were also treated with actinomycin D (5 µg/ml for 2 hours at 37°C/5% CO2, Sigma-Aldrich) to transiently inhibit transcription prior to the addition of camptothecin to confirm the transcription-dependant formation of R-loops.

### Topotecan treatment of ARCA mouse models

In order to exacerbate the formation of R-loops and determine whether R-loop could form in post-mitotic tissues upon DNA damage accumulation, ARCA mouse models were treated with Topotecan (TPT) (Sigma-Aldrich), a water-soluble derivative of camptothecin for a period of 9 days. Topotecan was administered daily (approximately at 10:00 am) as an intraperitoneal injection following a brief anaesthesia using Isofluorane gas (2.5% at 3 L/min). Isofluorane is one of the safest methods recognised for rodents anaesthesia. For each genotype group, 3 mice were injected daily either with carrier solution (Sterile Injection Water BP, Pfizer) or TPT (2 mg/Kg/Day) and weight was monitored and recorded daily prior and 24 hours post injection. Animal weights were recorded prior, during, and after the treatment regimen. General health and behaviour of the mice was monitored daily until the end of the treatment when the animals were sacrificed to assess for R-loop formation, apoptosis levels and proliferation status.

### R-loop forming sequences (RLFS) prediction and DNA/RNA Immunoprecipitation (DRIP)

R-loop forming sequence (RLFS) model [37] was applied to predict the location of RLFSs in the mouse genome. RLFS region located downstream to the poly(A) signal (<500 bp) were selected for DRIP analysis (Figure S5). Two candidates genes, the *Pkd2l1*gene located in the HS44.2 locus on chromosome 19 and the *Foxo4* gene located on the X chromosome, were selected for DRIP analysis. Genomic DNA and RNA extraction from *Setx^+/+^* and *Setx^−/−^* mice testes, LCLs and HeLa cells were performed using the DNeasy Blood & Tissue Kit (Qiagen, USA) following the manufacturers' instructions. Extracts were sonicated (maximum voltage, constant output, microtip limit) for 3 min with 1 min incubation on ice within each min. 40 µl of Protein G beads (50∶50 slurry) (Millipore, Germany) was added to the extracts and pre-cleared. In a separate tube, 15 µg of anti-R-loop antibody (S9.6) and 40 µl of Protein G beads (50∶50 slurry) (Millipore, Germany) were added together and incubated at 4°C overnight on a rotating wheel to allow binding of antibody to beads. Mouse anti-IgG antibody and 40 µl of Protein G beads (50∶50 slurry) (Millipore, Germany) were also added together to serve as a non-specific control for our experiment. The next day, the tubes were centrifuged at 5400× g for 2 min to pellet the beads. The supernatant was then divided equally into 3 tubes- 1 was left untreated, 1 was treated with 200 U of S1 Nuclease (Promega, USA) and the other was treated with 10 U of RNAse H (New England BioLabs, USA). Extracts were incubated at 37°C for 2 h, heat inactivated for 20 min at 65°C then added on to the antibody-bound Protein G beads and incubated at 4°C overnight on a rotating wheel to allow binding of R-loops fragments to antibody-bound beads. The next day, the beads were pelleted at 5400× g for 2 min and the supernatant was discarded. The beads were washed once with the IP Wash Buffer 1 (20 mM Tris-HCl pH 8.1, 2 mM EDTA, 50 mM NaCl, 1% Triton X-100, 0.1% SDS), twice with the High Salt Wash Buffer (20 mM Tris-HCl pH 8.1, 2 mM EDTA, 500 mM NaCl, 1% Triton X-100, 0.1% SDS), once with the IP Wash Buffer 2 (10 mM Tris-HCl pH 8.1, 1 mM EDTA, 0.25 M LiCl, 1% NP-40, 1% Deoxycholic Acid) and twice with TE Buffer (20 mM Tris-HCl pH 8.0, 1 mM EDTA). The immunoprecipitates were incubated at 4°C on a rocker for 3 min between each wash. The nucleic acids were then eluted twice with 100 µl of Elution Buffer (100 mM NaHCO3, 1% SDS). 3 µl of Proteinase K (20 mg/ml) was then added to each sample and incubated at 55°C. The samples were then diluted to 400 µl with TE Buffer and 3 µl of Glyogen (20 mg/ml) (Roche, Switzerland) was added. The nucleic acids were extracted using 400 µl of Phenol Chloroform (1∶1) and vortexed for 1 min. The samples were centrifuged at 16, 000× g for 5 min and the aqueous phase containing the nucleic acids were retained. These were extracted again using 400 µl of Chloroform under the same conditions. 2.5× of 100% EtOH was added to the samples and these were incubated at −80°C for 20 min. The samples were centrifuged at 16, 000× g for 20 min and the supernatant was decanted. The pellet was washed with 70% EtOH, centrifuged at 16, 000× g for 20 min and the supernatant was decanted. The pellet was air-dried and resuspended in EB Buffer (Qiagen, USA).

### PCR analyses from DRIP assay

Primers for the predicted RLFS of mouse HS44.2 region were mHS44.2 1F and mHS44.2 1R. Primers for the predicted RLFS of mouse Foxo4 region were mFoxo4 F and mFoxo4 R. RLFS regions are detailed in Figure S5. PCR cycling conditions were as follows: 35 cycles, initial denaturation at 95°C for 3 min, denaturation at 95°C for 45 sec, annealing at 60°C for 45 sec, extension at 72°C for 1 min, with a final cycle and extension of 7 min at 72°C. The PCR product size for the HS44.2 and Foxo4 regions were 226 bp was 357 bp respectively. See Figure S3 for list of primers.

### Senataxin immunoprecipitation

Immunoprecipitation of senataxin protein was performed as previously described [23].

### RT-PCR analysis for senataxin gene expression

Total RNA was isolated from 35-day-old wild type and knockout mice testes using the RNeasy mini kit (Qiagen, USA) according to the manufacturer's protocol. RNA concentrations were determined by UV spectrophotometry using a Nanodrop ND-2000 (Thermo scientific, USA) and cDNA was made from 1 µg of purified RNA using Super Script III (Life technologies, USA) according to the manufacturer's protocol. Gene expression analysis was performed by PCR in a 2720 Thermal Cycler (Applied Biosystem, USA). Reactions (25 µl) contained 14.5 µl of sterile water, 300 ng of cDNA template, 1× PCR Buffer II (Roche, Switzerland), 2.5 mM MgCl_2_ (Roche, Switzerland), 20 µM dNTPs, 1 µM of each primer, and 5 µl of AmpliTaq Gold DNA Polymerase (Roche, Switzerland). The primer pairs used for gene expression analysis are described in Figure S3. Amplification was for 30 cycles and cycling conditions were as follows: denaturation for 5 min at 95°C for 30 sec, annealing at 65°C for 30 sec, elongation for 1 min at 72°C followed by a final extension step of 7 min at 72°C. PCR reactions were separated on 2% TAE agarose gels and visualised using Ethidium bromide and GelDoc system (Biorad, USA).

## Supporting Information

Figure S1 **Lack of R-loops formation in** ***Atm^−/−^*** **,** ***Aptx^−/−^*** **and** ***Tpd1^−/−^*** **brain and cerebellar sections.** **A.** Specificity of the R-loop (S9.6) antibody. Serial testes sections from *Setx^−/−^* animals were either pre-incubated with RNAse H (1 hour at 37°C) or left untreated and subsequently immunostained for R-loops. As expected a reduction of R-loop fluorescence intensity is visible in RNAse H-treated samples thus confirming the specificity of the R-loop antibody. **B.** Histological sections of brain and cerebellum were stained for R-loops (Red) and TUNEL (Green). DAPI stained nuclei. Scale bar, 100 µm.
(TIFF)

Figure S2 **Knockdown of senataxin in HeLa cells using short interfering RNA (siRNA).** **A.** Following transfection of Control (Ctrl siRNA) and *SETX* siRNA, HeLa cells were immunostained for senataxin (SETX) and also processed for immunoblotting. As shown in panel A, a reduction in senataxin fluorescence signal was observed after *SETX* siRNA treatment. **B.** These data demonstrated the effective knock down of senataxin in HeLa cells via immunoblotting. **C.** Graph is plotted to show knockdown efficiency of *SETX*.
(TIFF)

Figure S3 **Primer pairs used for the genotyping of** ***Atm^−/−^*** **,** ***Setx^−/−^*** **,** ***Aptx^−/−^*** **,** ***Tdp1^−/−^*** **mice, PCR analyses for HS44.2 and Foxo4 regions from DRIP assay as well as RT-PCR.**
(TIFF)

Figure S4 **Senataxin expression in mouse tissues.** **A.** Senataxin immunoprecipitations from testes, brain, cerebellum, spleen and intestine were carried out to detect senataxin protein levels. A faint signal corresponding to senataxin protein was only detected in *Setx^+/+^* testes indicating lower levels of senataxin in the others tissues and/or weak sensitivity of the senataxin (Ab-1) antibody. **B.** RT-PCR analysis from testes, brain, cerebellum, spleen and intestine confirmed the lower levels of senataxin expression in brain, cerebellum, spleen and intestine compared to testes.
(TIFF)

Figure S5 **R-loop prediction and candidate genes selection.** **A.** An RLFS-containing region was found downstream of the *Pkd2l1* gene located in the HS44.2 locus. The predicted RLFS was found at the position Chr19: 44,221,689–44,221,838. **B.** In chromosome X, the RLFS located in *Foxo4* was found nearby a poly(A) signal. The predicted RLFS was found at the position ChrX: 98,456,266–98,456,499. For these genes, the predicted RLFS was located close to poly(A) signals (<500 bp) observed in mouse testes (by UCSC browser). Nucleotide sequence of the RLFS is in bold and primers are underlined.
(TIFF)
