# Correction: R-Loops in Proliferating Cells but Not in the Brain: Implications for AOA2 and Other Autosomal Recessive Ataxias

**DOI:** 10.1371/journal.pone.0105258

**Published:** 2014-08-06

**Authors:** The PLOS ONE Staff

The sixth author’s name is spelled incorrectly. The correct name is: Piroon Jenjaroenpun.
