# Supplementary figures and images for: R-Loops in Proliferating Cells but Not in the Brain: Implications for AOA2 and Other Autosomal Recessive Ataxias
Source: PLoS One. 2014 Mar 17;9(3):e90219. doi: 10.1371/journal.pone.0090219 (PMC3956458; doi:10.1371/journal.pone.0090219)

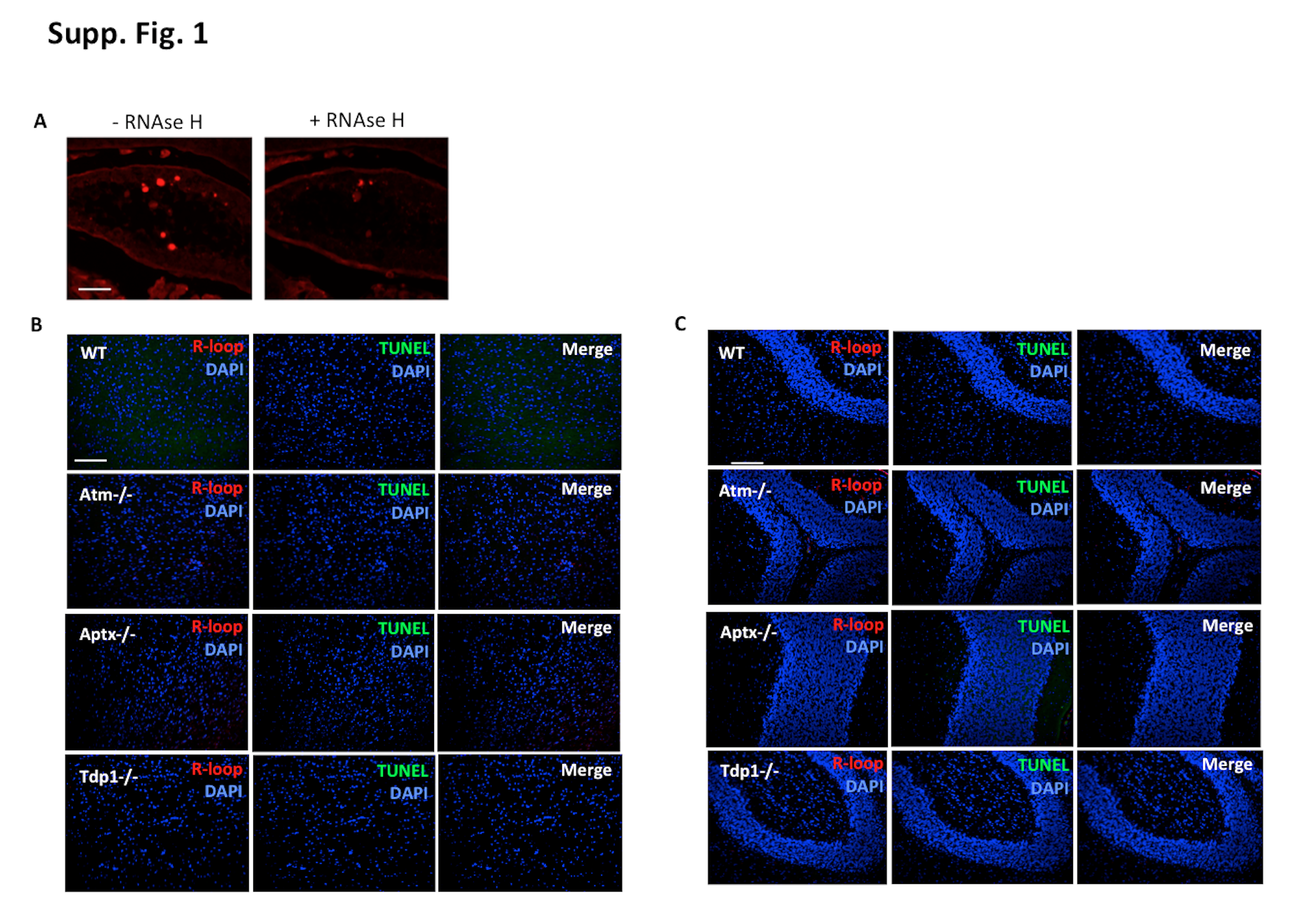

Supplement: Figure S1 — Lack of R-loops formation in Atm−/− , Aptx−/− and Tpd1−/− brain and cerebellar sections. A. Specificity of the R-loop (S9.6) antibody. Serial testes sections from Setx−/− animals were either pre-incubated with RNAse H (1 hour at 37°C) or left untreated and subsequently immunostained for R-loops. As expected a reduction of R-loop fluorescence intensity is visible in RNAse H-treated samples thus confirming the specificity of the R-loop antibody. B. Histological sections of brain and cerebellum were stained for R-loops (Red) and TUNEL (Green). DAPI stained nuclei. Scale bar, 100 µm. (TIFF) [file pone.0090219.s001.tiff]

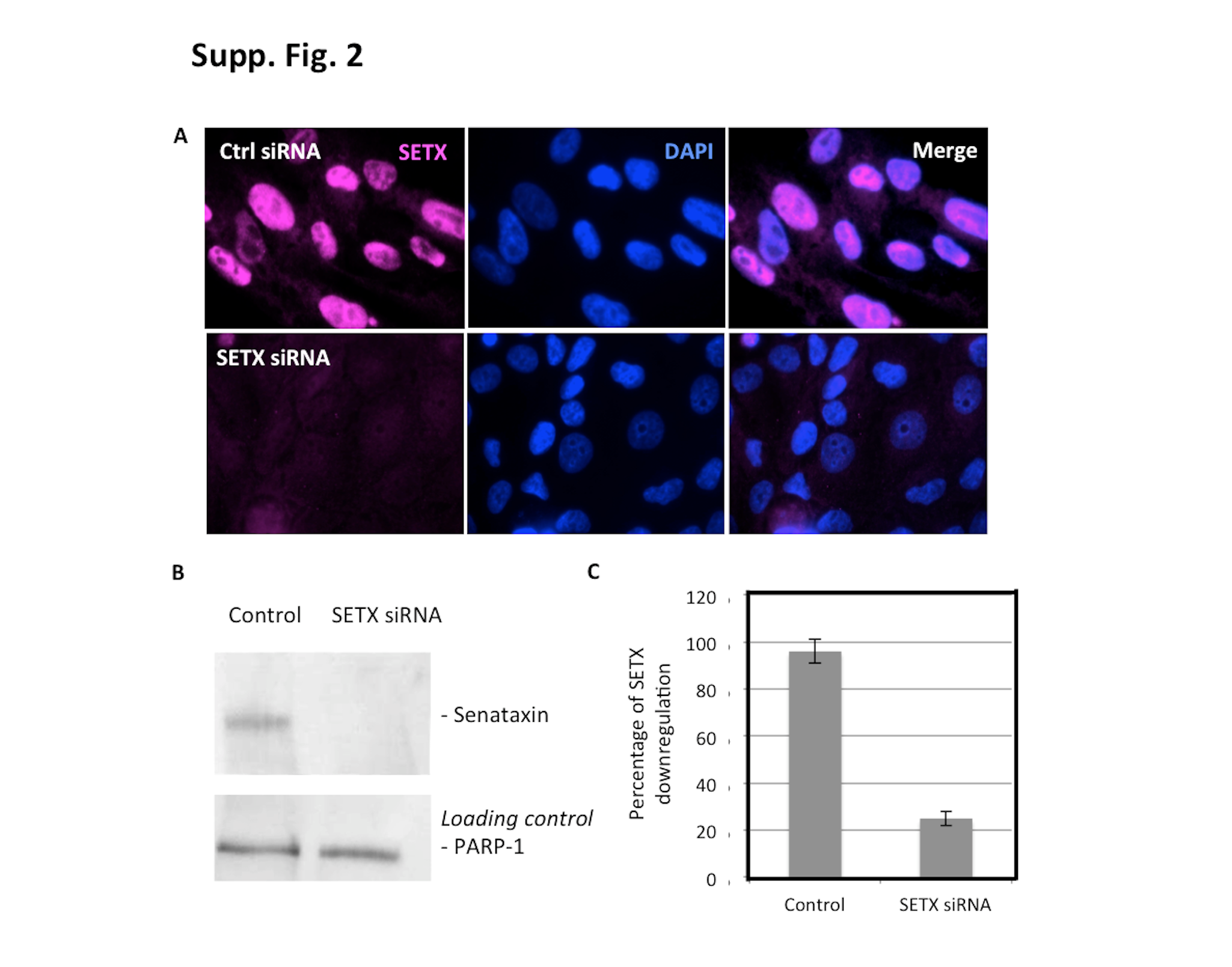

Supplement: Figure S2 — Knockdown of senataxin in HeLa cells using short interfering RNA (siRNA). A. Following transfection of Control (Ctrl siRNA) and SETX siRNA, HeLa cells were immunostained for senataxin (SETX) and also processed for immunoblotting. As shown in panel A, a reduction in senataxin fluorescence signal was observed after SETX siRNA treatment. B. These data demonstrated the effective knock down of senataxin in HeLa cells via immunoblotting. C. Graph is plotted to show knockdown efficiency of SETX. (TIFF) [file pone.0090219.s002.tiff]

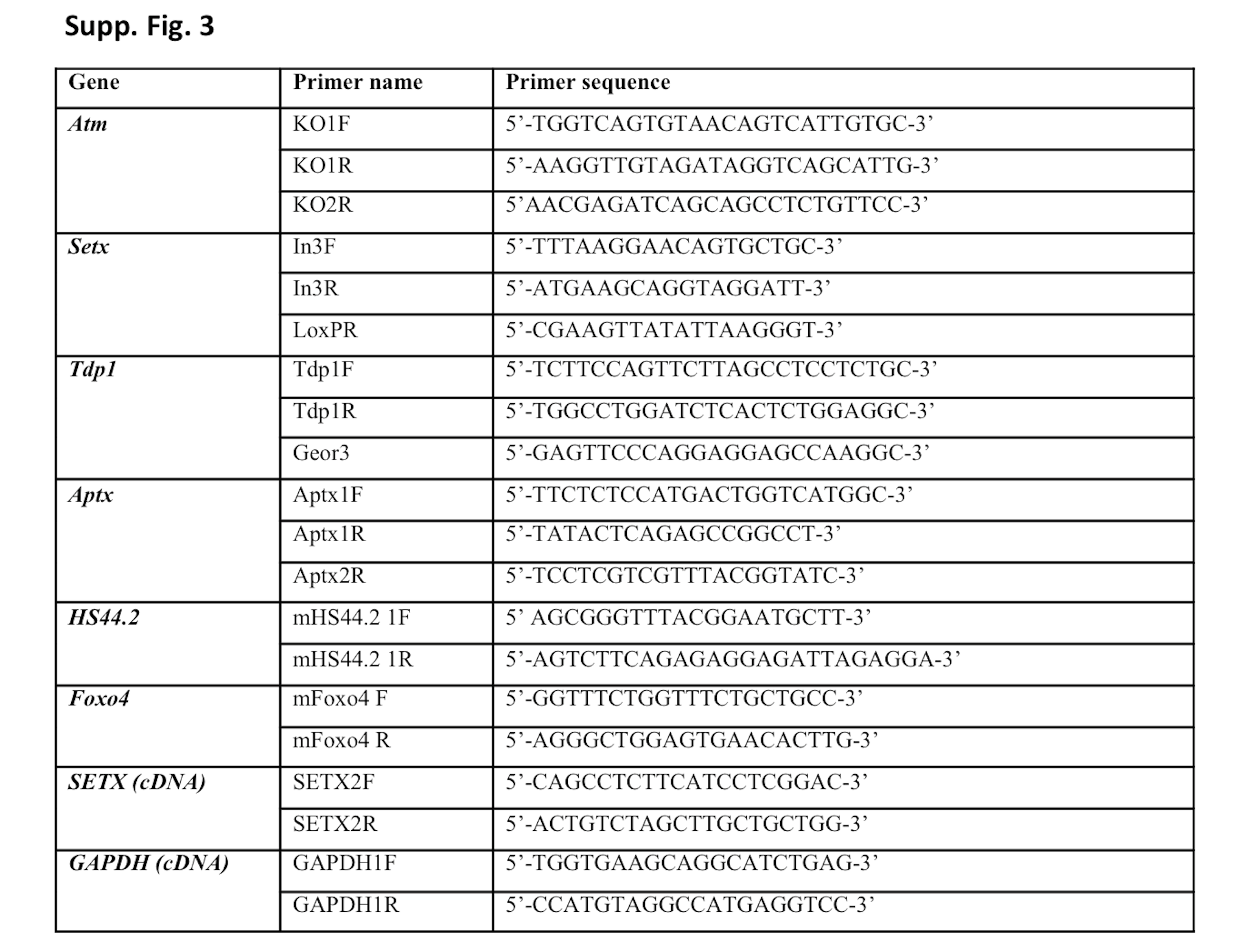

Supplement: Figure S3 — Primer pairs used for the genotyping of Atm−/− , Setx−/− , Aptx−/− , Tdp1−/− mice, PCR analyses for HS44.2 and Foxo4 regions from DRIP assay as well as RT-PCR. (TIFF) [file pone.0090219.s003.tiff]

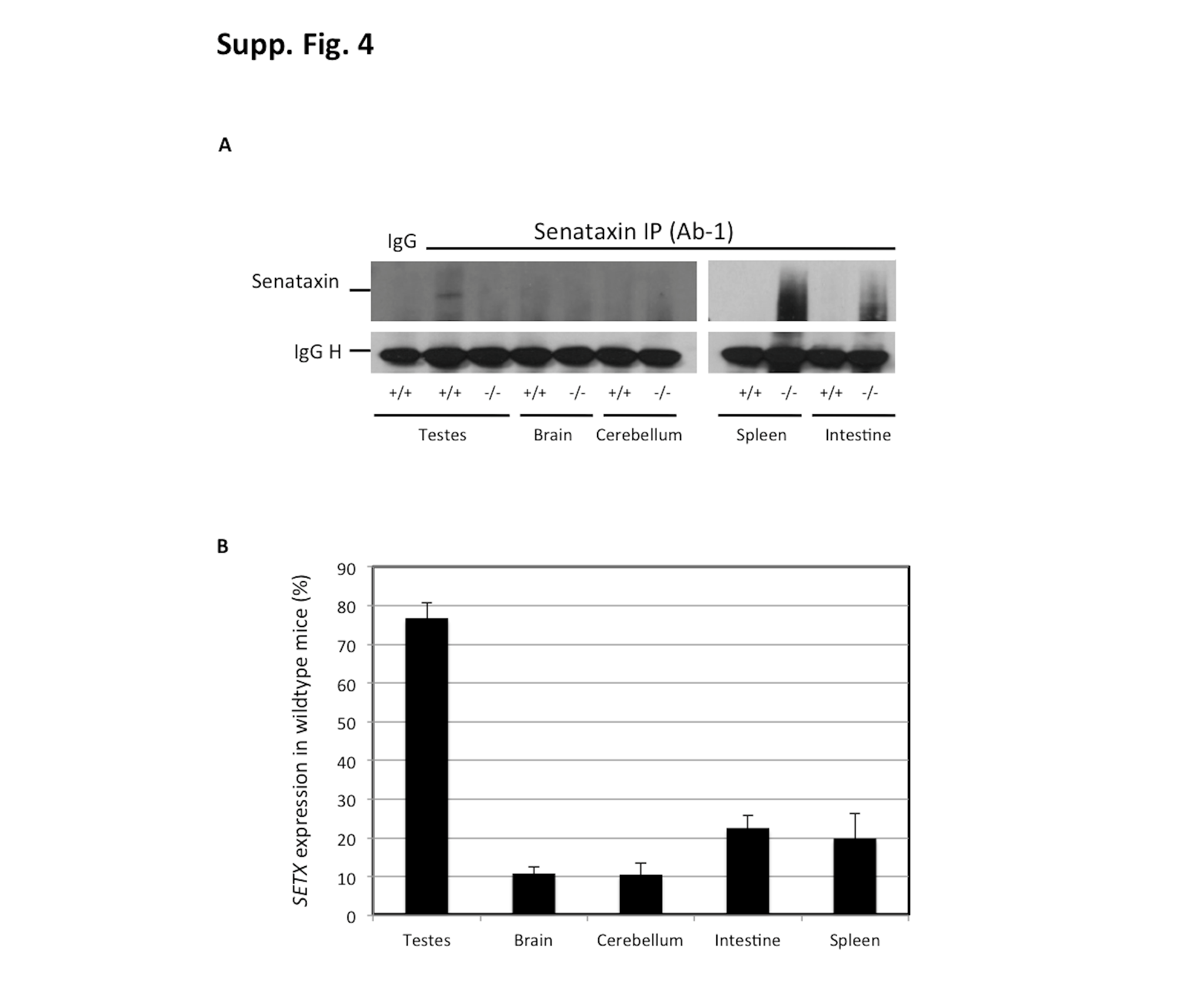

Supplement: Figure S4 — Senataxin expression in mouse tissues. A. Senataxin immunoprecipitations from testes, brain, cerebellum, spleen and intestine were carried out to detect senataxin protein levels. A faint signal corresponding to senataxin protein was only detected in Setx+/+ testes indicating lower levels of senataxin in the others tissues and/or weak sensitivity of the senataxin (Ab-1) antibody. B. RT-PCR analysis from testes, brain, cerebellum, spleen and intestine confirmed the lower levels of senataxin expression in brain, cerebellum, spleen and intestine compared to testes. (TIFF) [file pone.0090219.s004.tiff]

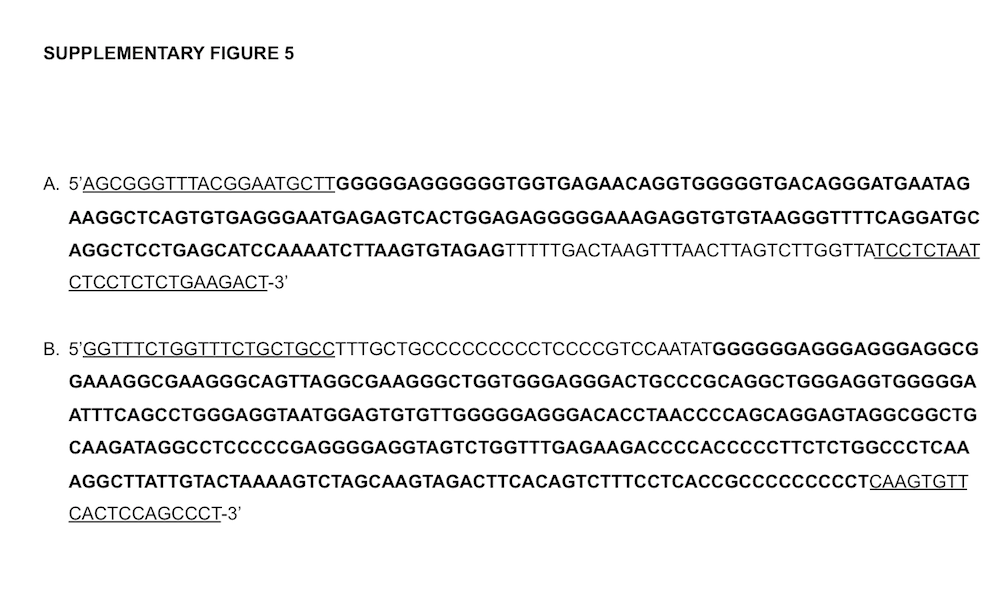

Supplement: Figure S5 — R-loop prediction and candidate genes selection. A. An RLFS-containing region was found downstream of the Pkd2l1 gene located in the HS44.2 locus. The predicted RLFS was found at the position Chr19: 44,221,689–44,221,838. B. In chromosome X, the RLFS located in Foxo4 was found nearby a poly(A) signal. The predicted RLFS was found at the position ChrX: 98,456,266–98,456,499. For these genes, the predicted RLFS was located close to poly(A) signals (<500 bp) observed in mouse testes (by UCSC browser). Nucleotide sequence of the RLFS is in bold and primers are underlined. (TIFF) [file pone.0090219.s005.tiff]
